# Integrating Patient-Generated Digital Data Into Mental Health Therapy: Mixed Methods Analysis of User Experience

**DOI:** 10.2196/59785

**Published:** 2024-12-16

**Authors:** Lauren Southwick, Meghana Sharma, Sunny Rai, Rinad S Beidas, David S Mandell, David A Asch, Brenda Curtis, Sharath Chandra Guntuku, Raina M Merchant

**Affiliations:** 1 Department of Emergency Medicine, University of Pennsylvania Perelman School of Medicine, 3600 Civic Center Boulevard, Philadelphia, PA, 19104, United States, 1-914-582-6995; 2 Center for Health Care Transformation and Innovation, Penn Medicine, Philadelphia, PA, United States; 3 Department of Computer and Information Science, School of Engineering, University of Pennsylvania Perelman School of Medicine, Philadelphia, PA, United States; 4 Department of Medical Social Sciences, Northwestern University Feinberg School of Medicine, Chicago, IL, United States.; 5 Department of Psychiatry, University of Pennsylvania Perelman School of Medicine, Philadelphia, PA, United States; 6 Department of Medicine, University of Pennsylvania Perelman School of Medicine, Philadelphia, PA, United States; 7 Intramural Research Program, National Institute on Drug Abuse, Baltimore, MD, United States

**Keywords:** digital data, social media, psychotherapy, latent Dirichlet allocation, LDA, mobile phone

## Abstract

**Background:**

Therapists and their patients increasingly discuss digital data from social media, smartphone sensors, and other online engagements within the context of psychotherapy.

**Objective:**

We examined patients’ and mental health therapists’ experiences and perceptions following a randomized controlled trial in which they both received regular summaries of patients’ digital data (eg, dashboard) to review and discuss in session. The dashboard included data that patients consented to share from their social media posts, phone usage, and online searches.

**Methods:**

Following the randomized controlled trial, patient (n=56) and therapist (n=44) participants completed a debriefing survey after their study completion (from December 2021 to January 2022). Participants were asked about their experience receiving a digital data dashboard in psychotherapy via closed- and open-ended questions. We calculated descriptive statistics for closed-ended questions and conducted qualitative coding via NVivo (version 10; Lumivero) and natural language processing using the machine learning tool latent Dirichlet allocation to analyze open-ended questions.

**Results:**

Of 100 participants, nearly half (n=48, 49%) described their experience with the dashboard as “positive,” while the other half noted a “neutral” experience. Responses to the open-ended questions resulted in three thematic areas (nine subcategories): (1) dashboard experience (positive, neutral or negative, and comfortable); (2) perception of the dashboard’s impact on enhancing therapy (accountability, increased awareness over time, and objectivity); and (3) dashboard refinements (additional sources, tailored content, and privacy).

**Conclusions:**

Patients reported that receiving their digital data helped them stay “accountable,” while therapists indicated that the dashboard helped “tailor treatment plans.” Patient and therapist surveys provided important feedback on their experience regularly discussing dashboards in psychotherapy.

## Introduction

An increasing amount of individual life is captured digitally, whether actively in the form of social media participation or passively in the form of software that tracks movement or telephone use or online engagement. There is a growing literature on how social media data like Facebook (Meta Platforms, Inc) wall posts or tweets (ie, posts on Twitter) and digital data (eg, search engine and smartphone metadata) offer insights into behavioral, social, and environmental factors that influence a person’s mental health and well-being [1-3]. Previous research found that social media trends and metadata can reveal and predict risk to mental health conditions, like depression and loneliness [4-6].

This emerging dataset offers a rich set of contextual information that could be beneficial within therapeutic encounters. Southwick et al [7] characterize how social media and digital data are used in psychotherapy from both the patient and mental health therapist perspectives. Patients reported periodically sharing social media posts with their therapist and how it provided important situational context to their discussion. Therapists shared experiences where reviewing social media posts and content was beneficial in delving into patient concerns and used social media platforms as exposure and homework for some patients. However, both patients and therapists underscored the importance of consent, transparency, and shared understanding to effectively use social media data in mental health therapy [7]. Similarly, Fisher and Appelbaum [8] reported that some psychotherapists incorporate parts of their patients’ Facebook feed in their care delivery and underscore how this access can provide additional insights into patients’ behaviors, moods, and other potential risk factors that often are not disclosed during mental health therapy. Furthermore, Hobbs et al [9] surveyed outpatient psychotherapists (n=115) and found that over two-thirds of outpatient psychotherapists reported viewing at least 1 patient’s social or electronic media as part of psychotherapy. Most of these psychotherapists (92%) reported that accessing electronic or social media improved their ability to provide effective treatment. Despite the promise demonstrated by the studies, most existing research relies on self-reported survey or interview data. Less is known about how patients and therapists experience the use of shared digital data during psychotherapy over time.

To address this gap in the literature, Merchant et al [10] conducted a randomized controlled trial (RCT; NCT04011540) investigating how receiving regular summaries of patients’ digital data (referred to as a digital health dashboard: Figure S1 in Multimedia Appendix 1) in psychotherapy impacted patient self-report health-related quality of life, depression or anxiety symptoms, and therapeutic alliance. The RCT systematically and passively collected social media and digital data from the patient weekly and distributed a digital data dashboard to both the patient and therapist prior to their weekly psychotherapy session for 2 months. The RCT found that incorporating the digital data dashboard into therapy did not statistically impact patient health-related quality of life, depression or anxiety symptoms, or therapeutic alliance over the 2-month study period [10]. Due to the null findings of the RCT, it is important to delve into the perspectives of both patient and therapist RCT participants to better interpret the results, especially since previous research reported that social media integration and discussion were useful and beneficial [7-9]. Understanding these experiences may allow for the refinement of this approach to improve its value. Using quantitative and qualitative methods, we captured RCT participants’ (eg, patients and their therapists) experience of regularly receiving digital health dashboards in psychotherapy.

## Methods

### RCT and Dashboard

RCT participants randomly assigned to the intervention arm regularly received a dashboard for 2 months. Digital dashboards (Figure S1 in Multimedia Appendix 1) were generated from consenting patients’ data from social media posts, online searches, and phone sensor data. The dashboards included 4 sections: late-night phone activity, physical movement, trending digital activity, and digital communication. Late-night phone activity was derived from AWARE (version 2; Open-source Context Instrumentation Framework For Everyone) and included the amount of time participants’ phone screens were on from midnight to 4 AM. We labeled the duration between midnight and 2 AM as nighttime and 2 AM to 4 AM as late-night activity. The physical movement section was derived using an AWARE pedometer plugin and included miles walked per week, days most walked, and days least walked. Trending digital activity was derived from status updates from Facebook and search queries from Google and YouTube. This section included the top 5 most frequent words from each platform within a 3-month period. The digital communication represented content from a digital source (eg, text message) that patients self-identified as wanting to include on the dashboard. These data were accompanied by responses to 2 prompts, “Why did you share this?” and “How did this make you feel?” (Figure S1 in Multimedia Appendix 1). Prior to receiving the first dashboard, both the patient and therapist received a brief 5-minute training video that described the dashboard, potential uses, and privacy or security risks.

### Ethical Considerations

All participants who were enrolled in the RCT (NCT04011540) conducted by Merchant et al [10] (ie, 57 patients and 47 therapists) were randomly assigned to the intervention arm (ie, regularly received a dashboard for 2 months) [10] and were asked to complete a postintervention debrief survey after the 2-month period. The survey data was de-identified and upon completion, participants received a US $50 gift card as compensation. The study protocol was reviewed and approved by the University of Pennsylvania’s Institutional Review Board (831246). The sample size allowed for thematic saturation. The study followed the Consolidated Criteria for Reporting Qualitative Research (COREQ) reporting guideline [11].

### Source Study: Data Collection

Two semistructured debriefing surveys (1 for patients and 1 for therapists) were developed based on a literature review of social media and digital data and psychotherapy, experience with the dashboard, and consumer service and experience questions like the net promoter score (NPS; Table S1 in Multimedia Appendix 1) [12]. Self-report surveys were administered via the Way to Health, Health Insurance Portability and Accountability Act (HIPAA) Compliant survey platform. Participants had the option to complete the survey via videoconference. Participants who selected an interview were conducted via videoconference with the first author (LS), a cis female researcher with more than 10 years of experience in qualitative interviewing. Participants who elected videoconference were asked survey questions verbatim and were audio-recorded. Interviews were approximately 30 minutes long.

### Statistical Analyses

We used descriptive statistics (eg, frequencies) to quantify study participants’ responses to close-ended questions. In line with the NPS scoring methods, scores were calculated by subtracting the “detractors” (scores of 0‐6) from “promoters” (scores of 9‐10) [12]. We conducted a post-hoc analysis of therapist NPS using pairwise comparisons.

Open-ended questions were analyzed using NVivo (version 10; Lumivero). A thematic analysis of responses was conducted using the constructivist paradigm. Thematic codes were developed through line-by-line reading of 20% of patient and therapist responses randomly by 2 independent readers (nonauthors). The independent readers met regularly to create a codebook and were supervised by a nonauthor. Each code was given an explicit definition and examples to ensure coding accuracy and to improve intercoder reliability. After the codebook was created, each reader then independently coded 5 deidentified transcripts. The κ score was above 0.75 and discrepancies in coding were resolved through a constant comparison approach [13].

Open-ended questions relating to dashboard experience were also analyzed using latent Dirichlet allocation (LDA) [14] to identify themes and keywords. LDA is a probabilistic topic modeling technique used to identify clusters of co-occurring words in text data. We used MALLET implementation [15] of LDA to generate topics in an unsupervised manner. We first removed noncontent words such as a, an, and is, using the Natural Language ToolKit (NLTK) Python library [16] along with the study-specific words such as dashboards. In addition, highly frequent words such as data and phone were also added to the stopwords list (Table S2 in Multimedia Appendix 1). We generated 3 sets of topics by varying the number of topics to 3, 4, and 5 with α=.5 and β=.01. Two coauthors (LS and MS) qualitatively examined the coherence of topics to select the best set and picked the final set of 3 topics.

## Results

### Study Sample

Of the 104 intervention participants, 96.1% participated in the debriefing survey or interview from December 2021 to January 2022. Our sample includes 56 patients and 44 therapists. Of which, most (n=89, 89%) were selected to complete the debrief via survey form and 7 patients and 4 therapists elected to complete the survey via videoconference (n=11, 11%). The study sample’s sociodemographics were similar to that of the total RCT sample [10]. Patients’ mean age was 31 years; 89.2% (n=50) were women and 83.9% (n=47) had been in therapy for more than a year. Therapists were mostly female (n=36, 81.8%) and White (n=36, 81.8%), who had a master’s degree (n=33, 75.0%). They worked in mostly private practices (n=31.8, 70.4%) and practiced cognitive behavioral therapy (n=14, 31.8%), eclectic psychotherapy (n=8, 18.2%), psychodynamic (n=5, 11.3%), family systems (n=4, 9.0%), family systems (n=4, 9.0%), and “other” forms of therapy (n=13, 29.5%)

### Descriptive Analyses

Most patients and therapists (n=48) described their experience with the digital health dashboard as “positive” (Table 1). Overall, most participants felt “very comfortable*”* sharing digital data and “very comfortable*”* reviewing and discussing the dashboard (Table S3 in Multimedia Appendix 1).

**Table 1.** Closed-end debriefing questions.

	Patient (n=56), n (%)	Therapist (n=44), n (%)
**On average, when did you discuss the dashboard in session?**		
Beginning	19 (34.5)	24 (54.5)
Middle	8 (14.5)	11 (25)
End	6 (10.9)	5 (11.4)
Prefer not to answer	4 (7.3)	4 (9.1)
Other	9 (16.4)	0 (0)
No answer	10 (16.4)	0 (0)
**On average, who initiated the showing or discussing the dashboard in session?**		
Provider initiated	7 (12.7)	26 (59.1)
Patient initiated	8 (14.5)	1 (2.3)
Both initiated	18 (32.7)	12 (27.3)
Prefer not to answer	16 (10.9)	5 (11.4)
Other	7 (12.7)	0 (0)
**How would you describe your experience with the digital health dashboard?**		
Positive	23 (41.8)	25 (56.8)
Neutral	21 (38.2)	19 (43.2)
Negative	1 (1.8)	0 (0)
Prefer not to answer	11 (19.6)	0 (0)

Patients and therapists most often used the dashboard at the beginning of the therapy session (Table 1). A total of 60% (n=26) of therapists noted that they initiated showing or discussing the dashboard in session whereas 32% (n=18) of patients reported “both the patient and therapist” initiated showing or discussing the dashboard (Table 1). The therapists reported a 14 NPS when asked if they would recommend the dashboard to a colleague and 0 to a patient. A pairwise comparison was −38 for therapists. Patients reported a −22 when asked to recommend using a dashboard to a friend in psychotherapy.

### Thematic Analysis

The results from LDA and directed content analysis were organized into three themes (with nine subcategories): (1) dashboard experience (positive, neutral or negative, and comfortable); (2) perception of the dashboard’s impact on enhancing therapy (accountability, increased awareness over time, and objectivity); and (3) dashboard refinements (additional sources, tailored content, and ethics) (Table 2).

**Table 2.** Themes, subthemes, and illustrative quotes.

Theme and subtheme	Patient illustrative quotes	Therapist illustrative quotes
**Dashboard experience**		
Positive	*I think that being a part of this study has helped me realize the impact of my phone use on my anxiety, and served as a starting block to discuss this in therapy.*	*My experience has been positive. The weekly updates are easy to access and review. The information has been helpful to ensure specific topics and issues are reviewed in session.*
Neutral or negative	*I didn’t feel that the dashboard gave me much useful info.*	*Not much change across the weeks.*
Comfortable	*Nobody is 100% cool donating data to somebody. [The research institution] is trusted.* *I felt completely comfortable reviewing my own data.*	*No material was unpredictable.* *[Dashboard] data wasn’t invasive, and information was voluntarily shared and consented.*
**Perception of the dashboard’s impact on enhancing therapy**		
Accountability	*It’s been helpful to re-examine the time I spend on social media. I also appreciate the digital dashboard follow-ups showing me how much I move.*	*[The dashboard] enhanced our session by giving my patient and me some talking points and ways to gauge how well the sessions were working to benefit their mental health as well as addressing some ways we can make the session more productive.*
Increased awareness over time	*[The dashboard helped me think] about therapy on non-therapy days started preparing me to think about that what I wanted to discuss in therapy that week.* *I found that my anxiety improved by seeing my therapist regularly and I noticed an extreme increase in symptoms whenever I chose to use social media.*	*The dashboard provided an opportunity to directly address material that was submitted, that was concerning. Although these are issues that are already being addressed in sessions, it provided some insight into how this client sometimes uses maladaptive and/or attention seeking ways to get comfort, validation, etc.* *There were several instances where material regarding [client] safety was presented on the dashboard. This led to safety assessments and exploration of some of the deeper emotions that this client was experiencing when stressed.*
Objectivity	*I enjoyed seeing the information and I would try to make sure I spent less time on my phone during the night.*	*Patient gained insight into their digital behavior and made choices about how to modify it when it becomes problematic.* *It was helpful to have data regarding a client’s phone usage other than self-report to increase interventions related to sleep hygiene, avoidance behavior, and increased behavioral activation.*
**Dashboard refinements**		
Additional sources	*As new platforms emerge, like Clubhouse, it’s worth exploring additional ways to capture other forms of digital data, such as voice dictation in the case of Clubhouse and videos in the case of TikTok.* *Add breakdown of apps used during those hours and give a percentage - like screen time on iPhone.*	*I think the dashboard did a good job of covering most of the areas that would be useful. Social media is definitely one place that offers a lot of information, as well as Google searches. Maybe in the future there could be an option for them to link an account that they think they use often.*
Tailored content	*Breakdown of that late night usage maybe a little more in depth when it comes to Facebook posts.*	*Certain words like drugs or things like designer drugs like ketamine.*
Privacy	*Digital data should be private, and therapy should operate on consent… I only participated in the study because I use a private browser for my more personal searches.*	*[Since digital] data is on the internet, it can never be totally secure, in the same way that paper data in a locked box in an office is, for example. So anytime we are using digital data or social media when working with client, there is the risk of breaching confidentiality.*

### Dashboard Experience

#### Positive

Patients and therapists noted a variety of reasons why they had a positive experience with the dashboard. Patients used the words “helpful*,” “*made*,” “*positive*,” “*phone*,” “*time*,”* and “likeable*”* while therapists used, “session,” “understand*,”* and “use*”* (Table 3; Table S4 in Multimedia Appendix 1) most often. A patient said, *“*I think that being a part of this study has helped me realize the impact of my phone use on my anxiety and served as a starting block to discuss this in therapy*.”* A therapist noted, “The information has been helpful to ensure specific topics and issues are reviewed in session” (Table 2).

**Table 3.** Keywords in intervention patients’ and therapists’ responses to “positive” and “neutral” experience with the digital health dashboard.

	Top 10 words in positive experiences	Top 10 words in neutral experiences
**Therapist**		
Topic 1	use, one, review, late, health, used, anxiety, helped, media, and talked	information, neutral, may, data, sessions, anything, between, personally, mentioned, and neither
Topic 2	discuss, information, sleep, concerns, useful, hygiene, able, liked, like, and patient	see, hard, helpful, useful, access, use, mind, found, work, and super
Topic 3	session, understand, social, data, clients, know, let, sessions, next, and navigate	week, find, helpful, vague, therapy, challenging, several, weeks, telehealth, and effectively
**Patient**		
Topic 1	made, positive, helpful, emotional, late, honest, words, understood, therapy, and YouTube	information, accurate, info, think, look, seem, find, fairly, helpful, and provide
Topic 2	phone, time, using, seeing, mental, tried, hours, movement, helps, and negative	feel, interesting, enough, interact, reveal, imagine, list, excite, less, and lot
Topic 3	like, able, health, think, changed, screentime, weeks, social, throughout, and held	useful, always, like, thing, keyword, liked, unhelpful, improved, know, and utilized

#### Neutral or Negative

Patients who expressed “neutral” experiences noted that the dashboard did not bring new or emerging topics into the therapy session. Most used words were, “information*,” “*feel*,”* and “useful*,”* while therapists’ said, “information*,” “*week*,”* and “vague*”* (Table 3). One patient noted a “negative” experience and said the dashboard was unhelpful and thought some of the dashboard data were “very inaccurate.”

#### Comfortable

Patients most frequently used words like “easy*”* and “fine*”* and therapists used words like “easy*,” “*information*,”* and “use*”* (Table 4; Table S4 in Multimedia Appendix 1) when describing their comfort reviewing and discussing the dashboard. When asked to elaborate on their comfort level sharing data, a patient said, *“*I felt completely comfortable reviewing my own data.” Another patient elaborated on how they liked seeing their digital data be used for a “better purpose.” A therapist shared, “[The dashboard] data wasn’t invasive, and information was voluntarily shared and consented.”

**Table 4.** Keywords in intervention patients’ and therapists’ responses to their comfort level reviewing and discussing the dashboard in mental health therapy.

	Top 10 words in comfort level reviewing the dashboard	Top 10 words in comfort level discussing the dashboard
**Therapist**		
Topic 1	easy, access, navigate, concerns, want, consented, know, review, view, and order	easy, clients, good, information, want, rapport, bad, bring, feel, and shared
Topic 2	information, week, feel, data, shared, felt, using, see, surprised, and need	discussing, comfortable, patient, open, fairly, session, presented, week, talk, and issue
Topic 3	client, use, clients, several, prior, able, involved, new, link, and study	client, discuss, concerns, hard, time, sure, depends, data, weekends, and with
**Patient**		
Topic 1	easy, time, review, fine, information, difficult, okay, know, showing, and use	things, talking, session, conversation, nothing, way, pretty, uncomfortable, little, and neutral
Topic 2	comfortable, felt, helped, talk, see, Facebook, nothing, discuss, easier, and every	discuss, therapist, something, want, bring, fine, talk, therapy, talked, and generally
Topic 3	reviewing, info, data, sharing, looking, texted, everything, gets, sleeping, and therapist	comfortable, sharing, like, discussing, anything, started, easy, feel, everything, and difficult

### Perception of the Dashboard’s Impact on Enhancing Therapy

#### Accountability

Both therapists and patients agreed that introducing data sources during therapy allowed patients to be more accountable for *“*their social media usage and honesty in appointments.*”* A patient stated that:

*It’s been helpful to re-examine the time I spend on social media. I also appreciate the digital dashboard follow-ups showing me how much I move*.

Similarly, another patient remarked how the dashboard:

*made me more aware of my social and emotional well-being. I like being held accountable for my social media account*.

A therapist said that using the dashboard “gave me some information on client sleep patterns that were relevant to our session.*”*

#### Increased Awareness Over Time

Since patients and therapists received a weekly dashboard for 2 months, many noted how they increased awareness of specific behaviors over the study period. Specifically, patients described that the data increased their self-awareness of their social and emotional well-being, stating how the dashboard facilitated thinking “about therapy on non-therapy days started preparing me to think about that what I wanted to discuss in therapy that week.” Sharing digital data helped patients recognize excessive technology usage patterns and allowed patients to examine the relationship between social media use and mood. A patient said, *“*I found that my anxiety improved by seeing my therapist regularly and I noticed an extreme increase in symptoms whenever I chose to use social media*.”* Another patient said,

*I’ve found that when I’m down my therapist isn’t just a text away, so I tend to post on Facebook. So, when my posts [are] shared my therapist has the opportunity to see my thoughts at the time I was having issues versus me recounting events at the end of the week.*

Therapists also appreciated the additional information. One noted:

*There were several instances where material regarding [client] safety was presented on the dashboard. This led to safety assessments and exploration of some of the deeper emotions that this client was experiencing when stressed*.

### Objectivity

The dashboard provided several modules highlighting objective data like time spent on one’s phone late at night (eg, midnight to 4 AM) and average miles walked per week (Figure S1 in Multimedia Appendix 1). A patient elaborated on how they

*found myself trying to become more aware of my digital technology use and has been a theme that I’ve discussed quite a bit in therapy. I think that being a part of this study has helped me realize the impact of my [late-night] phone use on my anxiety and served as a starting block to discuss this in therapy.*

Another patient shared, “I enjoyed seeing the information and I would try to make sure I spent less time on my phone during the night.” Therapists noted that the dashboard helped identify progress or specific concerns in a more objective way than self-reported disclosures. Specifically, a therapist revealed that a *“*patient gained insight into their digital behavior and made choices about how to modify it when it becomes problematic*.”* Similarly, another therapist said, “It was helpful to have data regarding a client’s phone usage other than self-report to increase interventions related to sleep hygiene, avoidance behavior, and increased behavioral activation*.”*

### Dashboard Refinements

#### Additional Sources

Participants provided recommendations to refine and improve the dashboard. Patients wanted to include more digital and social media platforms such as TikTok, more control of what type of information is shared from each data source, and a web interactive dashboard. Patients highlighted “As new platforms emerge, like Clubhouse, it’s worth exploring additional ways to capture other forms of digital data, such as voice dictation in the case of Clubhouse and videos in the case of TikTok*.”*

#### Tailored Content

Patients suggested additional metrics and filters to be added. For instance, a patient said they wanted a “breakdown of that late night usage maybe a little more in-depth when it comes to Facebook posts.” Therapists also provided suggestions such as more detailed Google searches, modules that highlight salient terms such as “drugs, gambling,” functionality that allows for communication between therapist and patient directly through the app, linking medical charts or electronic portal imaging devices and access to patient phone metadata (ie, how long patient has phone calls).

#### Privacy

When asked about ethical considerations, therapists expressed concerns about their patients’ data privacy and safety. One therapist noted:

*[Since digital] data is on the internet, it can never be totally secure, in the same way that paper data in a locked box in an office is, for example. So anytime we are using digital data or social media when working with client, there is the risk of breaching confidentiality.*

They also offered strategies to combat privacy concerns and decrease the risk of personal health information breaches by using HIPAA-compliant platforms and avoiding unencrypted applications (ie, FaceTime [Apple]). Informed consent on how data is used was underscored by both patients and therapists. One patient shared,

*digital data should be private, and therapy should operate on consent...I only participated in the study because I use a private browser for my more personal searches.*

## Discussion

### Principal Results

This mixed methods analysis provides insight on patients’ and therapists’ experience regularly receiving and incorporating dashboards in mental health therapy. Using LDA and directed content analysis, we identified 3 themes and 9 subcategories to characterize the RCT participant experience. We found that the two methods provide complementary strengths; LDA offers automated topic discovery and highlights keywords while the directed content analysis allows for nuanced interpretations. To our knowledge, this is the first study to capture both patient and therapist experiences using the same dashboard over a period of time. Our findings must be interpreted with the understanding that participants were those who had, in the underlying study, already consented to use these data in therapy. Individuals who enrolled in this study may be inherently different from the larger groups of individuals enrolled in mental health therapy.

Given that previous work by Merchant et al [10] found that a digital data dashboard into therapy did not statistically impact patient self-report health-related quality of life, depression or anxiety symptoms, or therapeutic alliance over the 2-month study period the interviews provide additional insight into the RCT null findings. The surveys revealed that most patients and therapists described their experience with the digital health dashboard as positive or neutral (Table 1). The proportion of neutral responses is noteworthy and may relate to dashboard differences by patient. Participants also shared several ways in which they perceived the dashboard to enhance therapy (Table 2). Most themes centered around awareness, objectivity, and accountability and future research should incorporate additional patient-centered validated measures such as self-awareness and patient activation to accurately capture the dashboard’s use. Indeed, patients and therapists were comfortable using the dashboard themselves and reported that the dashboard helped them stay “accountable.” Yet, in every case, they were less comfortable recommending the same approach for a friend or colleague. This is noteworthy as recommending a service or product is a hallmark metric for commercial success [12].

### Comparisons With Prior Work

A prominent finding was that patients enjoyed seeing trends over time and having this longitudinal data in the short term may have facilitated behavior changes such as spending less time on social media or on their phone at night. Therapists noted how the late-night module provided objective data to potentially tailor interventions. For instance, a therapist said, “It was helpful to have data regarding a client’s phone usage other than self-report to increase interventions related to sleep hygiene, avoidance behavior, and increased behavioral activation*.”* This quote exemplifies key measurement-based care (MBC) principles of being data-driven, objective, continuous monitoring, and enhanced personalization. This finding is similar to Hobbs et al [9] where psychotherapists noted a moderate or slight improvement in providing effective treatment due to accessing their patients’ digital data. Regularly receiving digital data metrics during the RCT can be compared to evidence-based practice of MBC, where client data are collected throughout treatment to individualize clinical decision-making [17-19]. MBC often involves regularly and systematically administering validated survey instruments such as symptom rating scales or ecological momentary assessments [18,19] to collect and monitor patient processes over time. In the RCT, rather than completing surveys over time, the digital data displayed on the dashboard were a proxy for key mental health and well-being domains, such as physical activity and time spent online from midnight to 4 AM (Figure S1 in Multimedia Appendix 1). Future work is needed to test if digital data metrics can be effective like MBC in mental health therapy.

Patients and therapists recommended improvements to the dashboard. Patients wanted more control of the data shared. This feedback is critical as there is limited research on regularly receiving digital data in psychotherapy. Yoo et al [20] found that patient autonomy and agency are critical when they asked clinical and human-computer interaction researchers about the design features of a clinician-facing tool for using insights from patients’ social media activity. In addition to design features, both patients and therapists also noted privacy concerns regarding social media data generally and specifically sharing it with one’s therapist [20]. This sentiment was underscored in previous research where patients undergoing mental health therapy thought sharing data with their therapist could be “creepy” [7]. This finding could be related to the national discussion of data privacy on social media platforms [21]. As such participants enrolled in the RCT were comfortable sharing and discussing data and due to the nature of the research, patients and their therapists both provided informed consent and the patients had full control of what type of data they shared and for how long.

### Limitations

Our study has several limitations. First, the RCT and survey were conducted during the COVID-19 pandemic, where most psychotherapy sessions occurred via videoconferencing and introducing the dashboard during this period might have been challenging. For instance, during the COVID-19 pandemic, therapists were managing substantially increased workloads and adding new tasks for some clients can create logistical challenges. Previous literature suggests that this issue can be mitigated by introducing a separate nonclinician professional or digital navigator to assist with technological concerns [22]. Although the American Psychological Association has shared that in 2022 “telehealth is here to stay” [23], telemental health therapy is not the most common modality of therapy. Previous research found that the transition to online telemental therapy affected therapeutic relationships as therapists shared feeling a lack of personal connection with their patients [24]. Second, most were conducted via survey, which limited follow-up questions. Third, participants enrolled in this study were willing to jointly review dashboards in psychotherapy and were heterogenous in terms of mental health conditions, time enrolled in therapy, educational attainment, race-ethnicity, and access to technology [10]. Fourth, given the iterative nature of qualitative analysis, we worked with trained coders to conduct the analysis and the coauthors reviewed the thematic analysis.

Future research is needed to determine if digital data in psychotherapy can improve patient clinical outcomes. If it is useful, as noted in the American Psychological Association’s “Guidelines for the Optimal Use of Social Media in Professional Psychological Practice,” training therapists on how to use social media data safely and ethically in therapy is paramount [25]. This training should also incorporate what therapists should look for in an app or digital tool such as data sharing, privacy, and confidentiality terms [26]. Similarly, the training should focus on how to introduce digital tools and obtain informed consent from the patient. To prepare both therapists and patients for using this novel data, clearly defined guidelines, policies, and best practices should be established and disseminated widely. In the context of receiving informed consent, privacy and confidentiality were highlighted by both stakeholders; future research should focus on a continuous process of informed consent [27].

### Conclusions

Our findings show that overall study participants enjoyed receiving a digital dashboard over time and elaborated on how the dashboard was used in therapy to hold them accountable. Therapists found the dashboard to provide insight and to be useful in augmenting care. Both groups highlighted ethical considerations and provided feedback on future dashboard design.

## Supplementary material

10.2196/59785 Multimedia Appendix 1 Digital data dashboard and additional information.

## Abbreviations

COREQ: Consolidated Criteria for Reporting Qualitative Research
HIPAA: Health Insurance Portability and Accountability Act
LDA: latent Dirichlet allocation
MBC: measurement-based care
NLTK: Natural Language Toolkit
NPS: net promoter score
RCT: randomized controlled trial
