# Supplementary material for: Integrating Patient-Generated Digital Data Into Mental Health Therapy: Mixed Methods Analysis of User Experience
Source: JMIR Ment Health. 2024 Dec 16;11:e59785. doi: 10.2196/59785 (PMC11683510; doi:10.2196/59785)
Supplement: Multimedia Appendix 1 [file mental-v11-e59785-s001.docx]

Figure S1. Sample digital health dashboard.

Table S1. Patient and therapist debriefing guide.

Table S2. Stop words used.

Table S3. Average comfort level and likelihood to recommend a dashboard.

**Figure S1.** Sample digital health dashboard.


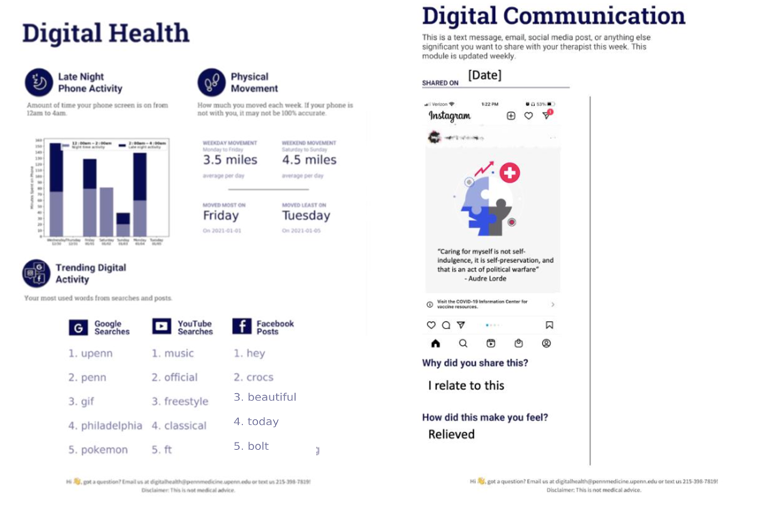


Figure caption: Digital dashboards were generated from consenting patients’ data from social media posts, online searches, and phone sensor data. Data were extracted from digital sources in several ways: through the API of social media platforms, via zip files upload for search data, and from the AWARE V2 platform. AWARE V2 is a free app which collects mobile sensor data from an individual’s phone. Using this data, dashboards were generated automatically and included four sections: late night phone activity, physical movement, trending digital activity, and digital communication. Late night phone activity was derived from AWARE V2 and included amount of time participants' phone screen was on from 12am to 4am. We labeled the duration between 12am to 2am as nighttime and 2am to 4am as late-night activity. The physical movement section was derived using an AWARE pedometer plugin and included miles walked per week, days most walked, and days least walked. Trending digital activity was derived from status updates from Facebook and search queries from Google and YouTube. This section included the top five most frequent words from each platform within a 3-month period. The Digital Communication represented content from a digital source (e.g., text message) that patients self-identified as wanting to include on the dashboard. This data was accompanied with responses to two prompts, “why did you share this,” and “how did this make you feel.”

**Table S1.** Patient and therapist debriefing survey guides. Closed-ended questions are italicized.

| **Patient** | Follow up / Probing Questions | | |
| --- | --- | --- | --- |
| Tell me about your experience in the study over the last 2 months? |  |  |  |
| What did you like about the study? | Please provide an example(s) |  |  |
| What didn't you like about the study? | Please provide an example(s) |  |  |
| *What was your comfort level downloading your data? 0-10, 0 is extremely uncomfortable, and 10 is extremely comfortable.* | Why did you pick that number? | How could we increase your comfort level? | How could we improve the data donation process? |
| How does telemental health therapy compare to in-person therapy? |  |  |  |
| Do you have any ethical concerns about using digital data or social media data in mental health therapy? |  |  |  |
| Any other comments or feedback for the research team? |  |  |  |
| **Patient - Intervention Specific** |  |  |  |
| Tell me about your experience with the digital health dashboard. |  |  |  |
| How would you describe your experience with the digital health dashboard? | How so? | Please provide an example |  |
| *Was the digital health dashboard positive, negative, or neutral?* | How so? | Please provide an example |  |
| *How often did you review the dashboard before each session?* |  |  |  |
| *How often did you and your therapist review or discuss the dashboard in session?* |  |  |  |
| *On average, who initiated the showing or discussing the dashboard in session?* |  |  |  |
| *On average, when did you discus the dashboard in session?* |  |  |  |
| Overall, how was the dashboard used in session? |  |  |  |
| What are some ways to improve dashboard? |  |  |  |
| What features would you have liked to see on the dashboard? | Remove features? | Anything else? |  |
| *What was your comfort level adding screenshots to the dashboard? 0 is Extremely Uncomfortable and 10 is Extremely Comfortable.* | Why did you pick that number? | How could we increase your comfort level? |  |
| *What was your comfort level reviewing the dashboard? 0 is Extremely Uncomfortable and 10 is Extremely Comfortable.* | Why did you pick that number? | How could we increase your comfort level? |  |
| *What was your comfort level discussing the dashboard? 0 is Extremely Uncomfortable and 10 is Extremely Comfortable.* | Why did you pick that number? | How could we increase your comfort level? |  |
| *How likely is it that you would recommend this dashboard to a friend in mental health therapy? 0 is very unlikely to recommend and 10 is very likely* | Why did you pick that number? | How could we increase your likelihood to recommend to a friend? |  |

| **Therapist** | Follow up / Probing Questions | |
| --- | --- | --- |
| Tell me about your experience participating in this study over the last 2 months? |  |  |
| How does telemental health therapy compare to in-person therapy? |  |  |
| What are the advantages about telemental health therapy? |  |  |
| What are the disadvantages about telemental health therapy? |  |  |
| Did you experience technical issues during the 2 months? |  |  |
| What was your experience completing the CIS checklist? |  |  |
| On average, how many minutes did you spend on completing the CIS checklist each week? |  |  |
| Lastly, considering the American Psychological Association (APA)’s Ethical Principles of Psychologists and Code of Conduct (Ethics Code), what ethical concerns do you have about using digital data or social media data in mental health therapy? |  |  |
| Any other comments or feedback for the research team? |  |  |
| **Therapist - Intervention Specific** |  |  |
| Tell me about your experience with the digital health dashboard? |  |  |
| *How would you describe your experience with the digital health dashboard?* | How so? | Please provide an example. |
| *Was the digital health dashboard burdensome, helpful, or other?* | How so? | Please provide an example. |
| *How often did you review the dashboard before each session?* |  |  |
| *How often did you and your patient review or discuss the dashboard during a session? On average, who initiated the showing or discussing the dashboard in session?* |  |  |
| *On average, when did you discus the dashboard in session?* |  |  |
| *Could you give me an example when the dashboard enhanced or augmented a therapy session?* | How often did this happen? |  |
| Could you give me an example when the dashboard detracted from or interfered with a therapy session? | How often did this happen? |  |
| Did the dashboard help with case formulation or case conceptualization? *Case conceptualization or case formulation 'is the clinician's collective understanding of the client's problems as viewed through a particular theoretical orientation; as defined by the biological, psychological, and social contexts of the client.' (John and Segal 2015) | How so? | Please provide an example. |
| What features would you have liked to see on the dashboard? | Remove features? | Anything else? |
| What are some ways to improve dashboard? |  |  |
| *What was your comfort level reviewing the dashboard? 0 is Extremely uncomfortable and 10 is Extremely comfortable.* | Why did you pick that number? | How could we increase your comfort level? |
| *What was your comfort level discussing the dashboard? 0 is Extremely uncomfortable and 10 is Extremely comfortable.* | Why did you pick that number? | How could we increase your comfort level? |
| *How likely would you be to recommend using a dashboard to a colleague? 0 is very unlikely to recommend and 10 is very likely to recommend.* | Why did you pick that number? | How could we increase your likelihood to recommend to a client? |
| *How likely would you be to recommend using a dashboard to a patient/client? 0 is very unlikely to recommend and 10 is very likely to recommend.* | Why did you pick that number? | How could we increase your likelihood to recommend to a colleague? |

**Table S2.** Stop words used.

| **Intervention specific questions** | **Group** | **Filtered terms** |
| --- | --- | --- |
| How would you describe your experience with the digital health dashboard? | Patient | [phone, data, time] |
|  | Therapist | [helpful, easy, information] |
| Neutral Experience with the digital dashboard | Patient | [information] |
|  | Therapist | NA |
| Positive Experience with the digital dashboard | Patient | [able, time, phone] |
|  | Therapist | [helpful, time, easy] |
| What was your comfort level reviewing the dashboard? | Patient | NA |
|  | Therapist | NA |
| What was your comfort level discussing the dashboard? | Patient | [discuss, therapist, comfortable] |
|  | Therapist | NA |

**Table S3.** Average comfort level and likelihood to recommend a dashboard.

| 0 is Extremely uncomfortable and 10 is Extremely comfortable | **Intervention**  **Patient** | **Intervention**  **Therapist** |
| --- | --- | --- |
|  | Average score (median) | |
| *What was your comfort level sharing digital data* | 7.4 (8) | . |
| *What was your comfort level reviewing the dashboard* | 8.8 (10) | 8.5 (10) |
| *What was your comfort level discussing the dashboard* | 7.5 (8) | 8.5 (9) |
| 0 is Extremely unlikely and 10 is Extremely likely |  |  |
| *How likely would you be to recommend using a dashboard to a colleague* | . | 7.6 (8) |
| *How likely would you be to recommend using a dashboard to a patient/client?* | . | 7.3 (7) |
| *How likely is it that you would recommend this dashboard to a friend in mental health therapy?* | 6.6 (7) | . |
